# Maxillary resection for cancer, zygomatic implants insertion, and palatal repair as single-stage procedure: report of three cases

**DOI:** 10.1186/s40902-017-0112-6

**Published:** 2017-05-25

**Authors:** Pietro Salvatori, Antonio Mincione, Lucio Rizzi, Fabrizio Costantini, Alessandro Bianchi, Emma Grecchi, Umberto Garagiola, Francesco Grecchi

**Affiliations:** 1 Department of Otorhinolaryngology-H&N Surgery, Humanitas San Pio X Hospital, Via F. Nava 31, 20159 Milan, Italy; 2 Department of Otorhinolaryngology, Ospedale Civile, Via Papa Giovanni Paolo II, 20025 Legnano, MI Italy; 3 Department of Oral Surgery, IRCCS Fondazione Ca’ Granda, Ospedale Maggiore Policlinico, Via F. Sforza 35, 20122 Milan, Italy; 4 Biomedical Surgical and Dental Sciences Department, Maxillo-Facial and Odontostomatology Unit, Fondazione Cà Granda IRCCS Ospedale Maggiore Policlinico, University of Milan, Milan, Italy; 5 Department of Maxillo-Facial Surgery, Istituto Ortopedico Galeazzi, Via R. Galeazzi 4, 20161 Milan, Italy

**Keywords:** Maxillectomy, Zygomatic implant, Tumour resection, Maxillofacial, Carcinoma, Maxillary reconstruction

## Abstract

**Background:**

Oronasal/antral communication, loss of teeth and/or tooth-supporting bone, and facial contour deformity may occur as a consequence of maxillectomy for cancer. As a result, speaking, chewing, swallowing, and appearance are variably affected. The restoration is focused on rebuilding the oronasal wall, using either flaps (local or free) for primary closure, either prosthetic obturator. Postoperative radiotherapy surely postpones every dental procedure aimed to set fixed devices, often makes it difficult and risky, even unfeasible. Regular prosthesis, tooth-bearing obturator, and endosseous implants (in native and/or transplanted bone) are used in order to complete dental rehabilitation. Zygomatic implantology (ZI) is a valid, usually delayed, multi-staged procedure, either after having primarily closed the oronasal/antral communication or after left it untreated or amended with obturator.

The present paper is an early report of a relatively new, one-stage approach for rehabilitation of patients after tumour resection, with palatal repair with loco-regional flaps and zygomatic implant insertion: supposed advantages are concentration of surgical procedures, reduced time of rehabilitation, and lowered patient discomfort.

**Cases presentation:**

We report three patients who underwent alveolo-maxillary resection for cancer and had the resulting oroantral communication directly closed with loco-regional flaps. Simultaneous zygomatic implant insertion was added, in view of granting the optimal dental rehabilitation.

**Conclusions:**

All surgical procedures were successful in terms of oroantral separation and implant survival. One patient had the fixed dental restoration just after 3 months, and the others had to receive postoperative radiotherapy; thus, rehabilitation timing was longer, as expected. We think this approach could improve the outcome in selected patients.

## Background

Major defects following maxillectomy for cancer include oronasal/antral communication, loss of teeth and/or tooth-supporting bone, and facial contour deformity. As a result, speaking, chewing, swallowing, and appearance are variably affected. Priority of restoration is focused on rebuilding the oronasal wall, by means either of flaps (local or free), either prosthetic obturator. Dental rehabilitation might follow by means of regular prosthesis, tooth-bearing obturator, and endosseous implants (in native and/or transplanted bone). Zygomatic implantology (ZI) has been first mentioned by Aparicio et al. in 1993 [1], then proposed by Brånemark [2] in order to overcome bone availability after maxillectomy. Commonly, this option is offered as delayed procedure after tumour resection. Later, ZI has been employed in non-neoplastic, severely atrophic maxilla [3–11]. The present paper is an early report of a relatively new, one-stage approach providing for tumour resection, palatal repair with loco-regional flaps, and zygomatic implant insertion in three patients. Advantages are concentration of surgical procedures, reduced time of rehabilitation, and patient discomfort.

## Case presentation

Three patients have been operated on for malignant neoplasms affecting the maxilla at the Legnano Hospital, Italy, and at the Humanitas San Pio X, Italy. Written informed consent was obtained from each patient, and the study protocol conformed to the ethical guidelines of the World Medical Association Declaration of Helsinki—Ethical Principles for Medical Research Involving Human Subjects. Surgical plan was based on tumour resection, palatal repair, and zygomatic implant insertion in view of fixed dental rehabilitation. CT scan for zygomatic bone evaluation was part of working up. No virtual planning of resection or of implant insertion was considered, and fixture placement was performed under direct vision, enhanced by simple resin guide simulating the resected dental arch. All patients were dentate (natively or after fixed restoration) and resulted partially dentate after tumour resection, so fitting class IIA defect classification, according to Pellegrino et al. [12]. Osteotomies were achieved with saw, burs, and piezosurgery. Frozen sections were obtained in order to demonstrate clean margins.

The zygomatic bone was adequately exposed. Implants from Noris Medical Ltd. (Nesher, Israel) were chosen. The working, threaded part of the implant is 13 mm long, while the remaining, fully smooth shaft has 4-mm diameter and variable length. In all, length ranges from 35 to 57.5 mm. Implant drilling was performed using both straight and angled handpieces. The fixtures were placed at 35 rpm for the 2/3 of the apical and manually for the most coronal 1/3 working part. Palatal-alveolar repair was attained with soft tissue, local flaps: these were also wrapped around the implants. In order to obtain a durable watertight seal between oral and nasal/antral cavities, implant uncovering and loading were planned to be deferred by 3 months.

CT scans and/or panoramic radiograph were taken to monitor implant healing.

Screw-retained fixed prosthesis was considered for teeth replacement.

### Patient no. 1

**Fig. 1:**
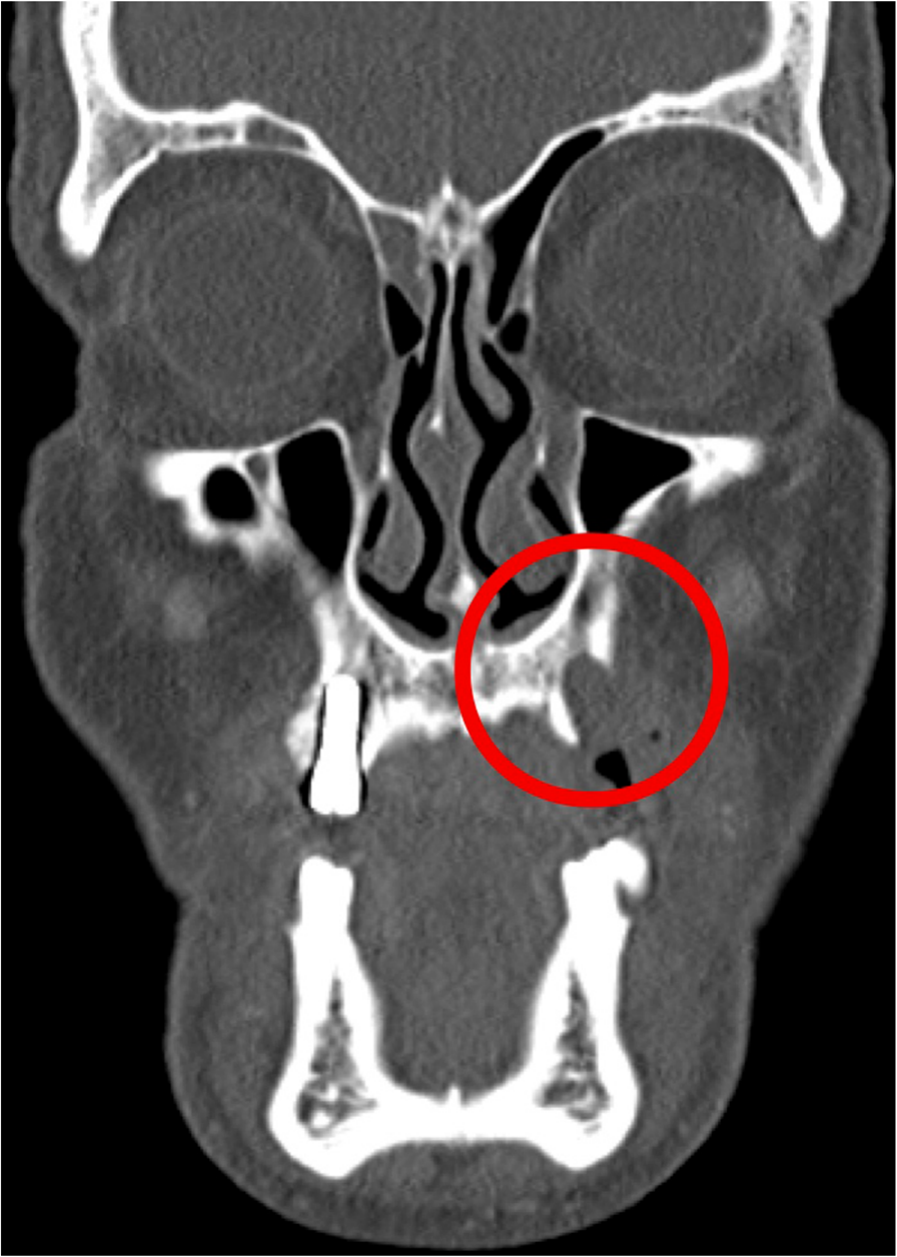
Pt no. 1. CT scan showing a radiolucent area involving the socket of tooth 24 and the surrounding bone

**Fig. 2:**
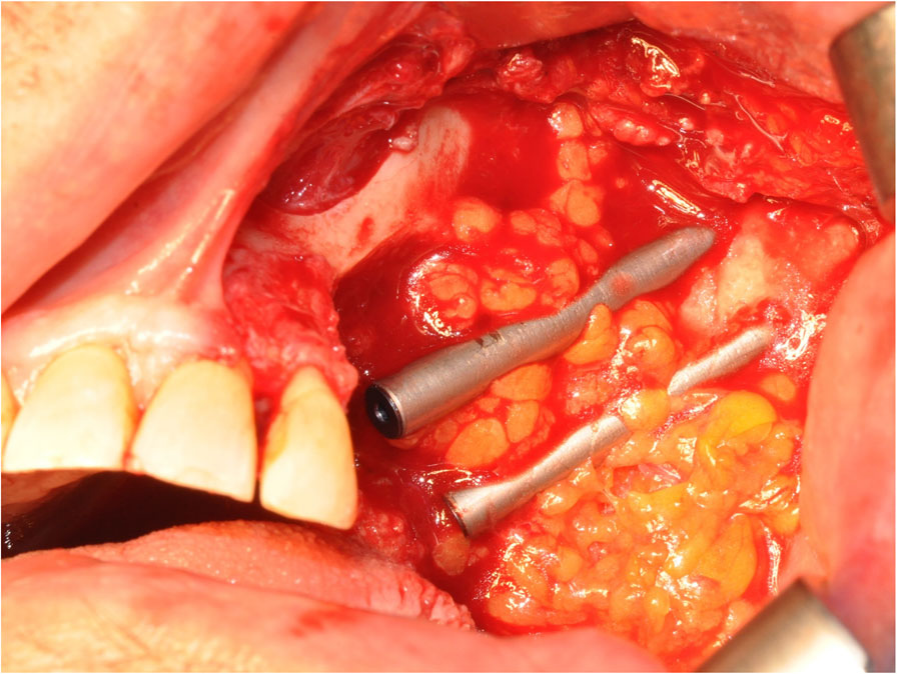
Pt no. 1. The buccal fat pad harvested and moved in order to repair the oroantral communication and to wrap the zygomatic implants

**Fig. 3:**
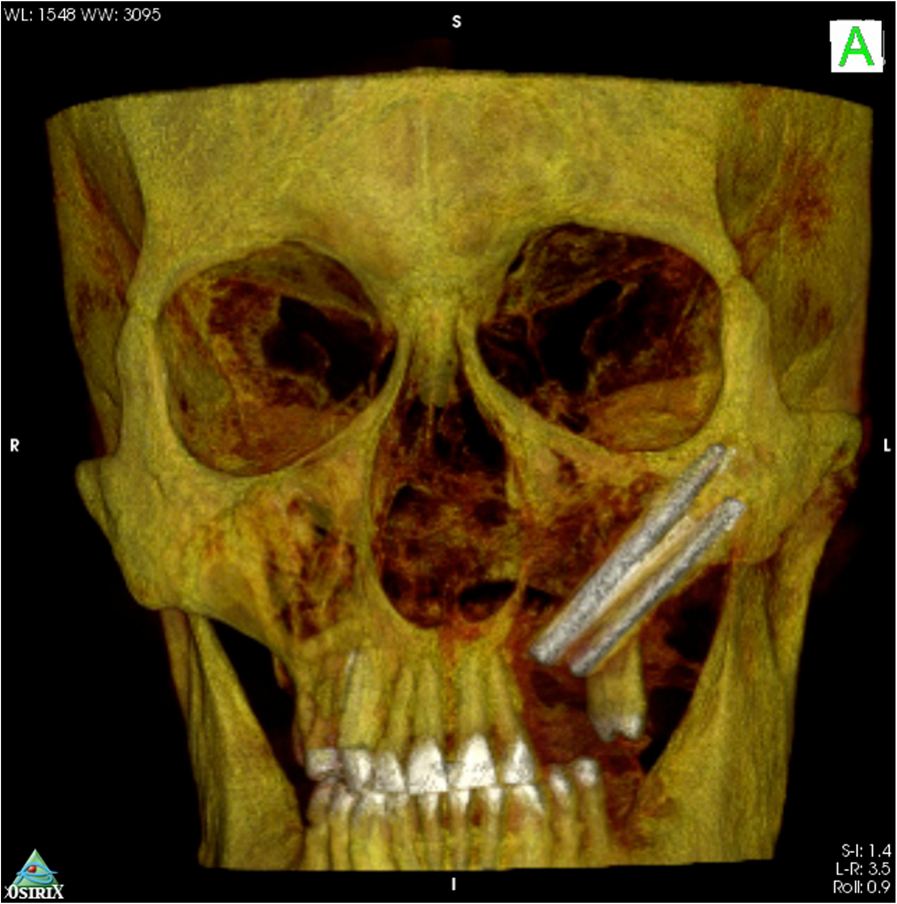
Pt no. 1. CT scan reconstruction showing zygomatic implant placement after maxillectomy

**Fig. 4:**
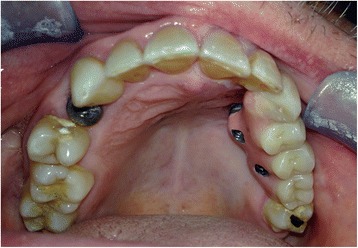
Pt no. 1. The final screw-retained prosthesis placed after 3 months

**Fig. 5:**
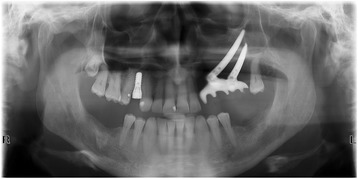
Pt no. 1. X-ray follow-up examination showing the final dental prosthetic rehabilitation

A 76-year-old gentleman suffering from lichenoid mucositis was operated on for verrucous carcinoma of the vestibular attached gingiva in the areas of 22 and 23, in 2013. The tooth 24 was missing, having been extracted elsewhere years before. Clear margins were obtained, and healing was uneventful. Then, the patient regularly attended follow-up examinations: on April 2015, a white, creamy discharge was noted from the gingiva covering the 24 socket. The gingiva was opened and the socket debrided. Histologic examination of the removed material was consistent with verrucous carcinoma. CT scan showed a radiolucent area involving the socket of 24 and the surrounding bone (Fig. 1). The neoplasm was staged T4 N0. The patient underwent partial maxillectomy involving the antral floor, the alveolar bone, and teeth 23 to 25. The tooth 26 had abnormal mobility, hence was extracted. Two zygomatic implants (40 and 42.5 mm, respectively) were placed into the malar bone. The buccal fat pad was harvested and moved to both repair the oroantral communication and wrap the implants (Fig. 2). The buccal mucosa was advanced over the buccal fat pad and implants and closed with single sutures. CT scan was taken after surgery (Fig. 3). Time was allowed for soft tissue healing, and after 3 months implants were uncovered, 45° abutments placed and a fixed, screw-retained prosthesis ended the rehabilitation (Fig. 4). To reduce direct loading on zygomatic fixtures, the prosthetic device was splinted mesially to 22 and distally to 27. After 1 year, the dental prosthetic restoration was unscrewed and zygomatic implant stability successfully checked (Fig. 5).

### Patient no. 2

**Fig. 6:**
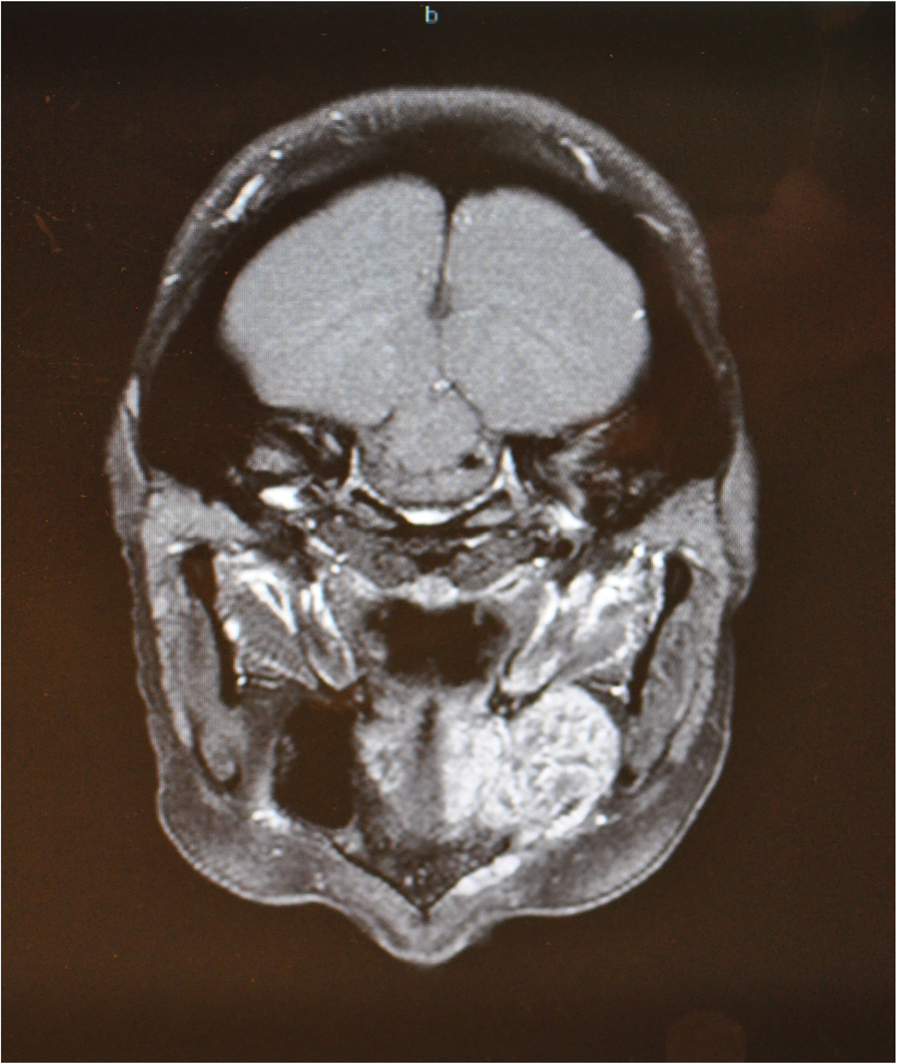
Pt no. 2. Transverse plane of the preoperative CT scan showing a radiopaque mass of the left maxilla

**Fig. 7:**
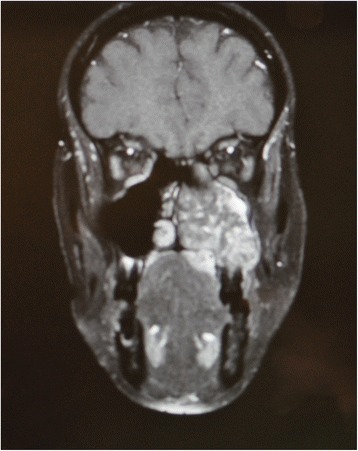
Pt no. 2. Coronal plane of the preoperative CT scan showing a radiopaque mass of the left maxilla

**Fig. 8:**
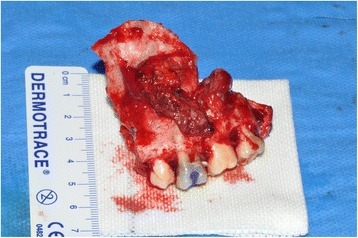
Pt no. 2. The extended portion of the left maxilla removed after maxillectomy

**Fig. 9:**
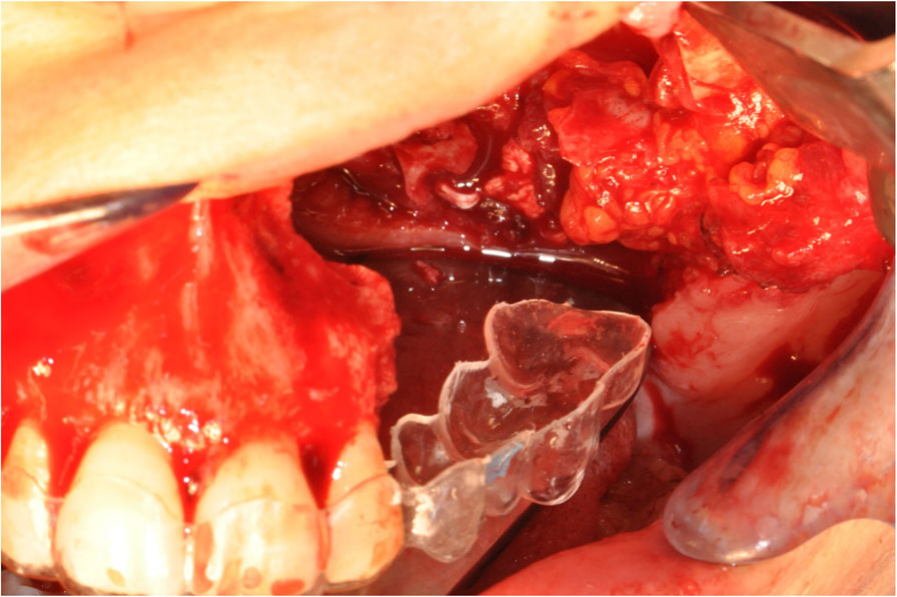
Pt no. 2. The prefabricated occlusal replica used for correct zygomatic implant emergence planning

**Fig. 10:**
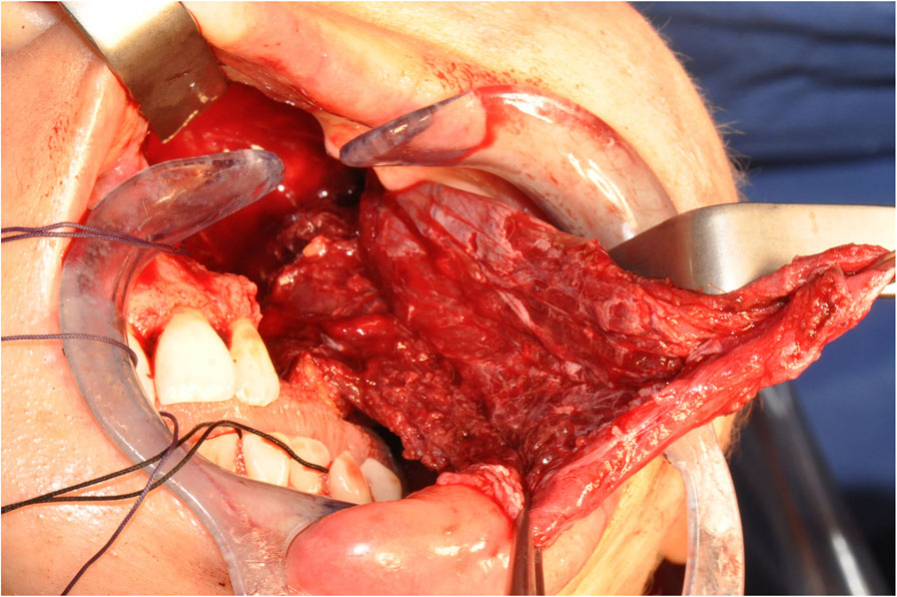
Pt no. 2. The left temporalis muscle raised and rotated in order to fill the defect and to wrap the zygomatic implants

**Fig. 11:**
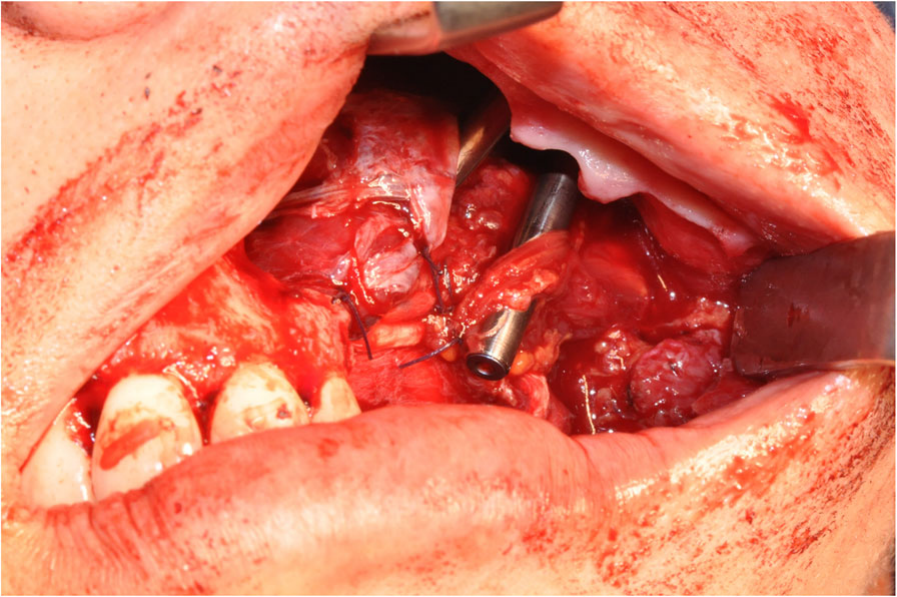
Pt no. 2. The fascial side of the left temporalis muscle stitched to the mucosal margin to separate the sino-nasal cavity from the oral one

A 43-year-old lady bearing an adenoid cystic carcinoma of the left maxilla was referred for treatment. Clinical and radiologic examination leaded us to stage the tumour T4 N0 (Figs. 6 and 7). The patient underwent left extended maxillectomy (Fig. 8). A prefabricated occlusal replica (Fig. 9) allowed the most correct insertion of two zygomatic implants (40 and 42.5 mm, respectively). Then, the left temporalis muscle flap was entirely raised and rotated to fill the defect and to wrap the implants (Fig. 10). The fascial side was stitched to the mucosal margins in order to separate the sino-nasal cavity from the oral one (Fig. 11). The postoperative period was uneventful, and care had been taken in order to contrast trismus since the surgery. The final pathologic report alerted against perineural invasion, and some spotted margins close to the tumour. These data, together with the tumour nature and extension at presentation, led to address the patient to receive a full course of adrotherapy. Regrettably, the latter treatment carried some important sequelae (radionecrosis in the pterygoid region and trismus, mostly antalgic) that forced to delay dental rehabilitation. However, hyperbaric oxygen therapy and sequestrectomy granted the complete healing of radionecrosis and trismus improvement: implant stability was checked during this in-office surgery and appeared fully satisfactory, so did CT scan imaging. Pathologic examination did not reveal any relapsing disease.

### Patient no. 3

**Fig. 12:**
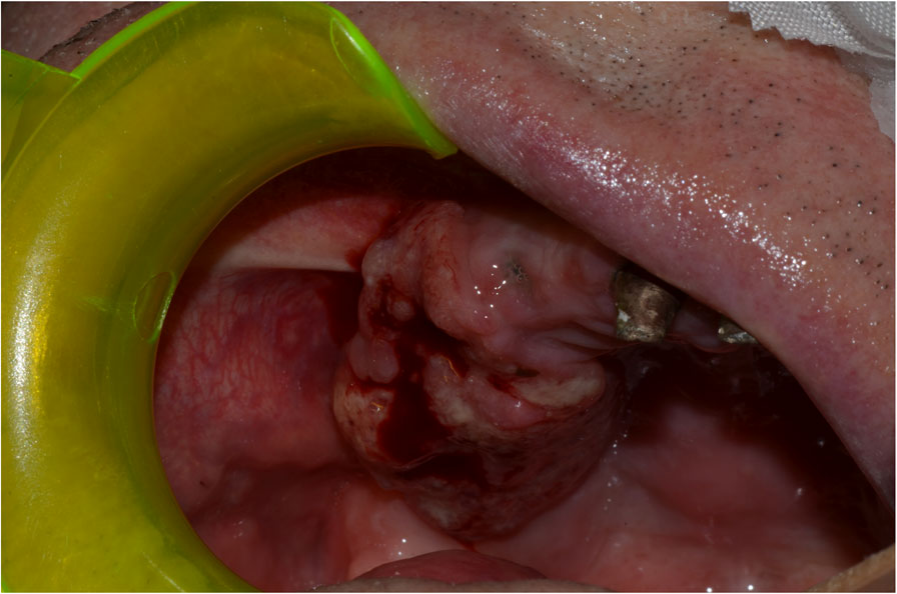
Pt no. 3. Ulcerated SCC of the right upper gingiva

**Fig. 13:**
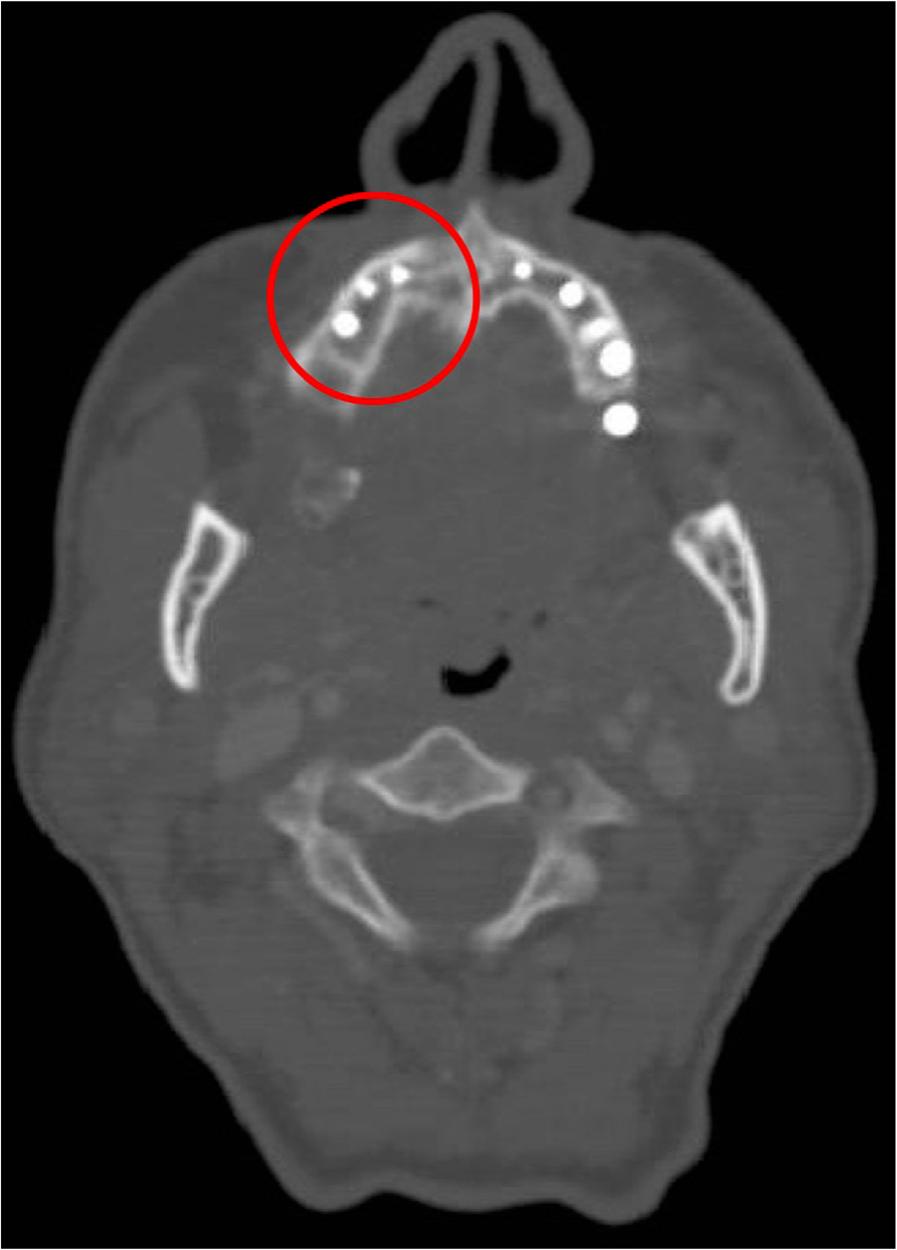
Pt no. 3. Preoperative CT scan

**Fig. 14:**
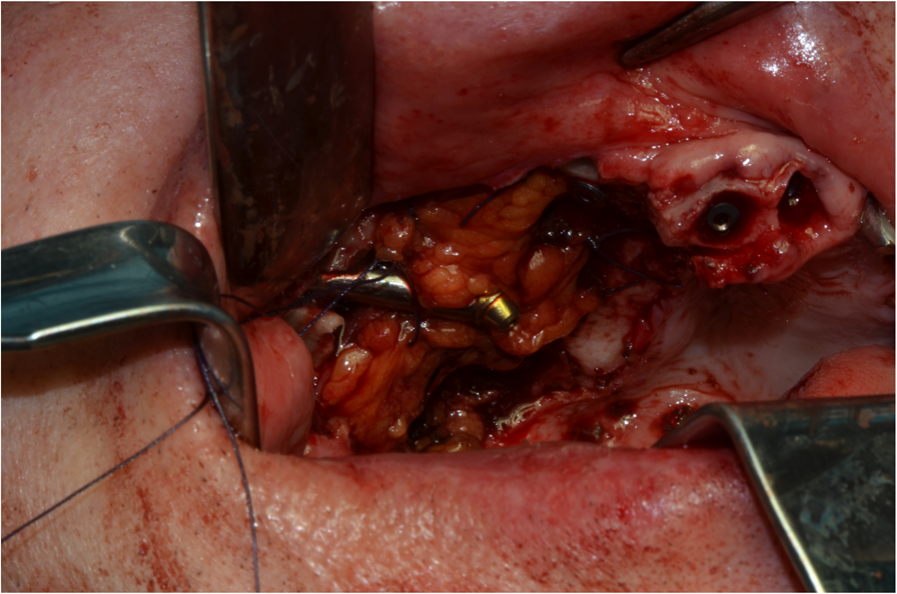
Pt no. 3. The zygomatic implant emerging in area of tooth 16 and surrounded by the buccal fat pad

**Fig. 15:**
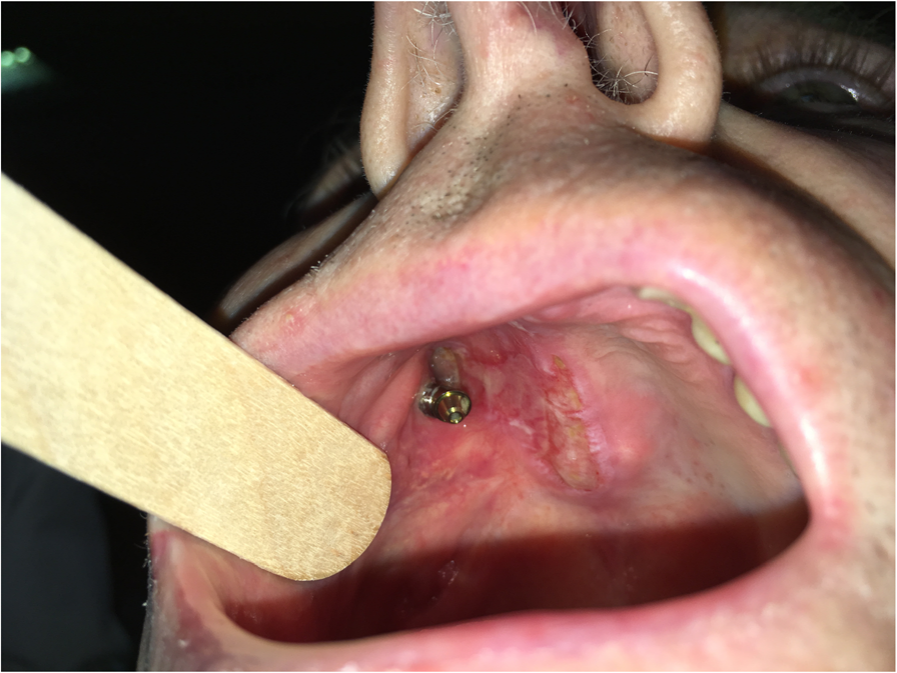
Pt no. 3. Intraoral view after 1 month

**Fig. 16:**
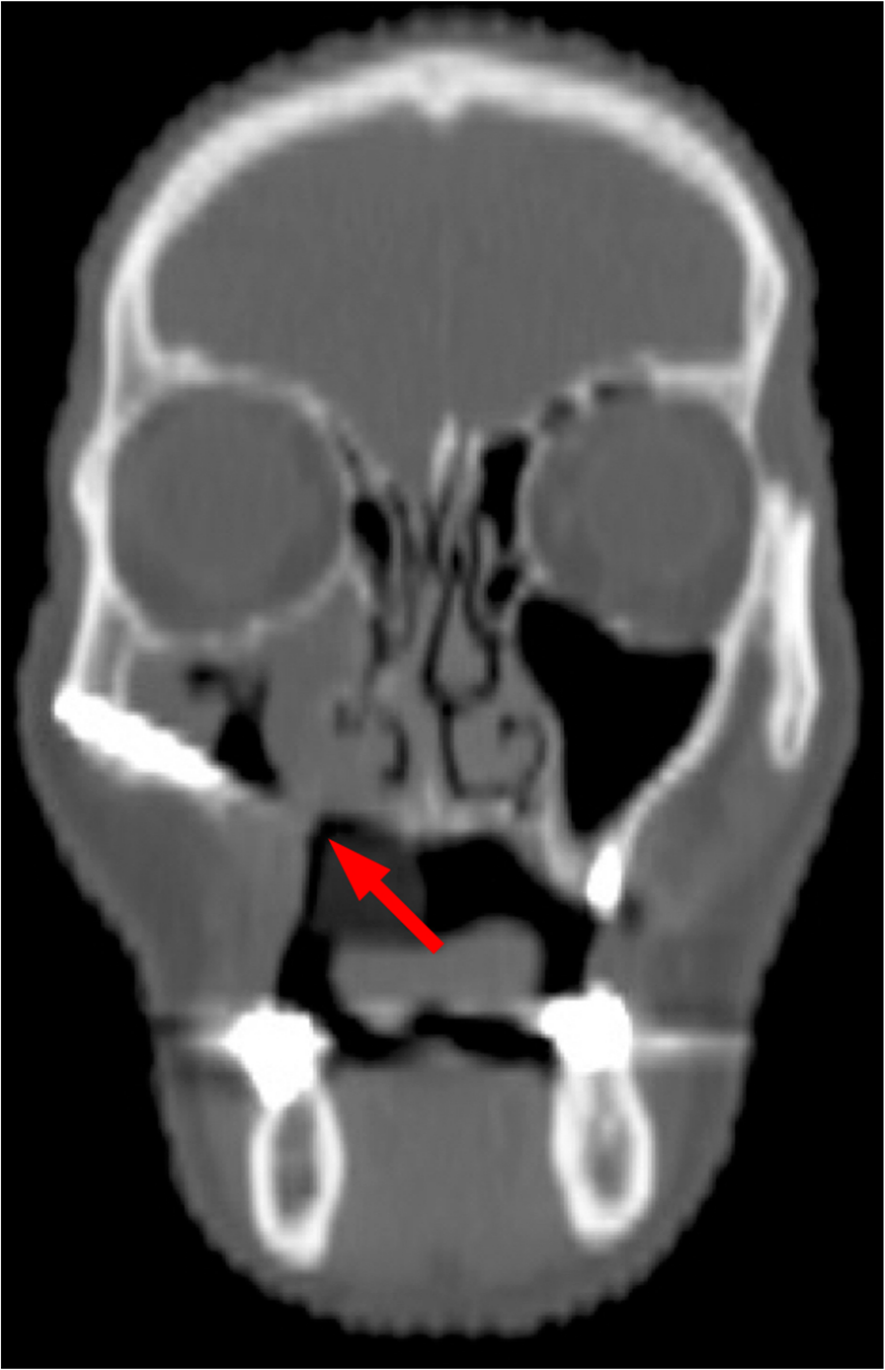
Pt no. 3. CT scan showing the optimal ZI insertion and the newly formed oroantral barrier

A 65-year-old gentleman suffering from squamous cell carcinoma of the upper gingiva underwent right partial maxillectomy (Fig. 12). The lesion showed have arisen around three endosseous implants placed years before in the teeth 13, 14, and 15 areas. The CT scan did not demonstrate frank bone involvement, neither neck node extension (Fig. 13) nor distant metastases. Consequently, a large oroantral communication derived from tumour ablation (that had to include the three implants); the fat pad flap preoperatively planned was judged adequate after harvesting and actually used to close the defect. Compromised teeth 11 and 21 were also extracted and immediately replaced by two standard implants. A third standard, tilted implant was posed in the 13 area. Finally, one zygomatic implant was inserted in order to emerge in the 16 area (Fig. 14). Postoperative course was complicated by limited suture dehiscence, without oroantral fistula, and spontaneous healing was then reached adopting a conservative treatment (Figs. 15 and 16). Pathologic examination demonstrated clear margins in the sinus mucosa, but bone invasion upstaged the patient from cT2 to pT4, and then, adjuvant radiotherapy was advised. Soft tissues were allowed to recover from radiation upshots and the prosthetic timing was subsequently scheduled.

### Discussion

Neoplasms of the maxilla often require extensive surgery and adjuvant treatments: as a consequence, quality of life might result as heavily impaired.

Reconstructive surgery (immediate or delayed) allows anatomic and basic functionality restoration following maxillary tumour resection. Actually, the most important goal has to be achieved—as earlier as possible— is the repair of the natural barrier between oral and nasal/antral cavities: options include free or local flaps and obturator.

Free flaps may either be harvested as single component, or as soft tissue and bone complex. Among the latter, fibula, iliac crest, and scapula are the most popular, with personal preference for the fibula flap. These composite auto-transplants allow both restoration of the oronasal/antral barrier and bone support for implants. Disease-related indications for composite free flaps include repair of large defects (2/3 of the palato-alveolar complex) and 3-D maxillary reconstruction. Their use implies large consumption of resources, yet patients’ survival is quite rewarding [13].

In contrast, local and regional flaps are less demanding, but their use is restricted to more limited palato-alveolar defects (up to the midline). The temporalis muscle is the workhorse for repairing such defects, while buccal fat pad has room in case of minor oronasal/antral communications [14]. When needed, adequate bone support may be set by secondary bone grafting.

Finally, prosthetic obturator is recommended when the above solutions cannot be available or are contraindicated: it requires adequate anchoring (residual dentition, standard implants, deep vestibular sulcus) and continuous servicing.

In our opinion, primary closure by flaps should be preferred over prosthetic obturator, as this approach makes the patients more comfortable and prosthesis-free, immediately and during his/her daily activity. Indeed, in all three patients, local flaps have performed well and led to successful immediate closure of the oroantral communication following tumour ablation. Seok et al. [14] advocate the application of 4-hexylresorcinol in order to accelerate and improve re-epithelialization.

Common belief stresses that follow-up in patients wearing obturator would be easier and safer than that in patients having surgical closure of the palate. In fact, possible local recurrence of the tumour could be detected early, yet benefit in survival of such a policy has not definitively proved. Moreover, modern imaging techniques could be at least as effective as inspection in revealing possible relapse at an early stage.

Nevertheless, some patients are or become more demanding about full or maximum recovery of the finest activities linked to chewing, phonation, deglutition, and aesthetics: in these cases, dental rehabilitation through implant-supported prosthesis might greatly help, the fixture(s) being usually inserted in native or grafted bone. Zygomatic implants could overcome the possible problem of lacking or poor-quality bone [2, 5, 12, 15–22]. In such patients, ZI is usually a delayed, multi-staged procedure, either after having primarily closed the oronasal/antral communication [12, 17, 19], either after left it untreated or amended with obturator [5, 16, 18]: the overall time from tumour treatment and final dental rehabilitation might require 1 year or more. Intuitively, interest has arisen in shortening this gap and we planned to move toward this direction.

The relatively innovative aspect of the present paper deals with the idea of challenging three different tasks in a single-stage procedure: resection of the tumour, closure of the oronasal/antral communication, and insertion of the zygomatic implants finalized to a fixed restoration. In few words, we tried to reach the best cost/benefit ratio.

Indeed, Pellegrino et al. [12] should be credited for the first reported case, even if not clearly evident from their paper (personal communication from Prof. C. Marchetti). The authors also proposed a new classification of rehabilitation-orientated maxillary defects: in our opinion, it deserves attention because of its clarity and effectiveness in orientating therapeutic options.

We were able to complete the above plan within the expected period of 3 months in patient no. 1, whose outcome is optimal after 1 year.

Supplementary advantage of ZI at the time of tumour resection is to give implants sufficient time to become osseointegrated before prospective radiotherapy course, then avoiding or minimizing its well-known negative impact on healing [23]. Actually, patient nos. 2 and 3 took some benefits from this policy.

In addition, applying a maxillary prosthesis in the early stages minimizes contraction of facial soft tissues [16].

We performed ZI under direct vision, enhanced by resin guide pointing landmarks. The procedure was somewhat easier than ZI in simply atrophic patients, as the resected bone allowed more room to vision and manipulation. On the other hand, the prepared flaps and the residual dentition could make things a bit more difficult than usual situations. Some authors advocate either general [24] or specific computer-aided surgery [12, 25], or surgical navigation [15, 26], for accurate, safe zygomatic implant installation. Undoubtedly, these are effective apparatuses, whose limitations are availability and operating costs. The pilot hole technique [27] and piezosurgery could offer similar advantages—at least in terms of safety—with lower costs.

Zygomatic implants are most suitable for immediate loading in reason of the high torque usually necessary for their insertion and consequent outstanding primary stability. However, we privileged the delayed loading to achieve and maintain an adequate seal between oral and nasal/antral cavities.

Long-term results of ZI are quite satisfactory. Brånemark [2] reported a 97% success rate in a series of 184 zygomatic implants inserted in 81 patients. Aparicio et al. [10] conducted a large review of zygomatic implant survival: success rates ranged 94.4 to 100%. Recently, Chrcanovic et al. [11] extended the analysis over 4556 zygomatic implants in 2161 patients: they found a noteworthy 12-year cumulative survival rate of 95.21%.

Despite the prosthetic aspects of the proposed technique are somewhat beyond the paper scope, some considerations appear obliged. Screw-retained, metal-core dental prostheses are popular, manageable devices allowing easy removal for fixture inspection and cleaning. An interesting point is that in patient no. 1, the interdental and inter-arch obligations lead to a double-cantilevered dental restoration, entailing a possible overload: to mitigate it prudently, mesial (to 23) and distal (to 27) splinting were conceived. Indeed, implant stability was preserved, as checked at regular clinical and X-ray follow-up examinations (Fig. 5).

Within reason, delayed ZI insertion in regard of radiotherapy and/or primary ablative surgery would have been more hazardous and difficult, if not impossible. In turn, fixed dental restoration would have been more demanding, more lasting, suboptimal, even not feasible. Concisely, immediate insertion of ZI at the ablative tumour time could be considered as a biological investment.

## Conclusions

In selected cases, maxillary resection, zygomatic implant(s) placement, and palato-alveolar repair through local flaps can be conducted as same-stage procedure. Advantages would include the following:

- Immediate closure of the oronasal communication
- Quick return to normal or near-normal feeding and phonation
- Wide access to bony segment receiving zygomatic implants
- Unnecessary bone grafting
- Short surgery time
- Reduced number of substantial interventions
- Short time-to-rehabilitation
- Reduced financial impact
- Valid functional results
- Excellent long-term performance of ZI

We intend to propose this approach and wish the results will be confirmed in large series.

## Abbreviations

Pt: Patient
ZI: Zygomatic implantology
